# Predictors of survival following extracorporeal cardiopulmonary resuscitation in patients with acute myocardial infarction-complicated refractory cardiac arrest in the emergency department: a retrospective study

**DOI:** 10.1186/s13019-015-0212-2

**Published:** 2015-02-24

**Authors:** Sang Jin Han, Hyoung Soo Kim, Hyun Hee Choi, Gyung Soon Hong, Won Ki Lee, Sun Hee Lee, Dong Geun You, Jae Jun Lee

**Affiliations:** 1 Division of Cardiology, Department of Internal Medicine, Hallym University Medical Center, Hallym University College of Medicine, 77 Sakju-ro, Chuncheon, 200-704 Republic of Korea; 2 Department of Thoracic and Cardiovascular Surgery, Hallym University Medical Center, Hallym University College of Medicine, 77 Sakju-ro, Chuncheon, 200-704 Republic of Korea; 3 Department of Urology, Hallym University Medical Center, Hallym University College of Medicine, 77 Sakju-ro, Chuncheon, 200-704 Republic of Korea; 4 Department of Anestheology, Hallym University Medical Center, Hallym University College of Medicine, 77 Sakju-ro, Chuncheon, Republic of Korea

**Keywords:** Acute myocardial infarction, Cardiac arrest, Extracorporeal circulation, Extracorporeal membrane oxygenation

## Abstract

**Background:**

This study aimed to identify the determinant factors for clinical outcomes and survival rates of patients with cardiac arrest (CA) concurrent with acute myocardial infarction (AMI) who underwent extracorporeal cardiopulmonary resuscitation (ECPR) using extracorporeal membrane oxygenation (ECMO).

**Methods:**

We retrospectively evaluated 37 patients admitted to our emergency department between January 2006 and August 2012 for AMI-induced CA treated with ECPR during ongoing continuous chest compressions.

**Results:**

Mean patient age was 61.4 ± 11.3 years, and 27 patients (73%) were men. Mean CPR time was 50.8 ± 35.4 min. Door-to-ECMO and door-to-balloon times were 84.4 ± 55.3 and 98.4 ± 56.8 min, respectively. Mean ECMO time was 106.4 ± 84.7 h; nine (24%) patients died within 24 h after ECMO initiation. Twelve (32%) patients were weaned off ECMO, seven (19%) of whom survived >30 days after ECMO removal; all except one had Cerebral Performance Category Grade 1. Of the patients who survived, 5 of them were able to be discharged. In multivariate analysis, statistical significance was only observed in door-to-ECMO time ≤60 min (OR, 6.0; 95% CI, 1,177–852.025; p = 0.033).

**Conclusion:**

We conclude that ECMO insertion within 60 min of the arrival of patients with AMI and CA at the ED appears to be a good option for maintaining myocardial and systemic perfusion, thereby increasing the survival rate of these patients.

## Background

Although prehospital management and early revascularization therapy have considerably contributed to enhance the survival rate of patients with acute myocardial infarction (AMI) concurrent with cardiogenic shock or cardiac arrest (CA), these are the most fatal complications associated with the high mortality rate of AMI [1-4]. Some reports have recently presented cases in which extracorporeal membrane oxygenation (ECMO) was successfully administered to patients with AMI concurrent with cardiogenic shock, thus enhancing their survival rate [5-9]. Moreover, the use of ECMO is increasing because, in combination with cardiopulmonary resuscitation (CPR), ECMO has superior efficacy compared to that of conventional CPR in the treatment of in-hospital CA cases due to various causes [10].

Since not much is known about the practice of extracorporeal cardiopulmonary resuscitation (ECPR) using ECMO in patients with AMI-induced CA, we conducted this study to identify the clinical outcomes of ECMO combined with ECPR as well as the factors influencing survival in patients with AMI and CA.

## Methods

### Patient enrolment criteria and pre-ECMO management

**Figure 1:**
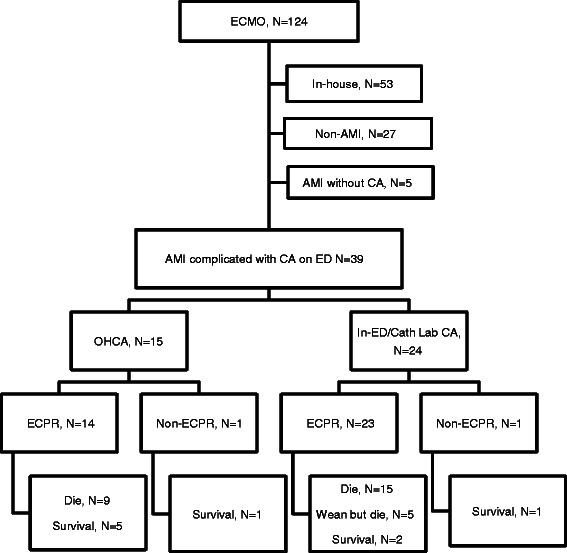
**Flow diagram of the study population and outcome.** ECMO, extracorporeal membrane oxygenation; AMI, acute myocardial infarction; CA, cardiac arrest; ED, emergency department; OHCA, out-of-hospital CA; Cath Lab, catheterization laboratory; ECPR, extracorporeal cardiopulmonary resuscitation.

ECMO was performed for 124 patients with acute heart failure or acute respiratory failure, for which conventional treatment was ineffective, between January 2006 and August 2012 in the 400-bed Hallym University Chuncheon Sacred Heart Hospital affiliated with Hallym University located in a small town with a population of 300,000. Among the 71 patients who received ECMO on the day of admission to the emergency department (ED), we conducted a retrospective study on 37 patients who received veno-arterial ECMO upon the recurrence of CA within 20 min after the return of spontaneous circulation (ROSC) or due to no signs of ROSC after >10 min of CPR following AMI-induced CA (Figure 1). All patients underwent ECMO during ongoing continuous chest compressions. CPR time was calculated by subtracting ROSC time from the total chest compression time. Patients with unwitnessed cardiac arrest, ongoing intracranial hemorrhage, or terminal cancer were excluded. This study was approved by the institutional review board of Hallym University Chuncheon Sacred Heart Hospital (IRB No. 2012-91).

### ECMO equipment

Three types of centrifugal pumps were used to deliver the ECMO: Capiox Emergency Bypass System® (Terumo, Inc., Tokyo, Japan) and Bio-pump® (Medtronic Inc., Minneapolis, MN, USA) were used from 2006 to May 2010; from June 2010 onward, a Centrifugal Rotaflow pump® (Maquet Inc., Hirrlingen, Germany) was used in most patients. Depending on patient size, we used 17–21 Fr arterial cannulae (DLP®, Bio-Medicus, Medtronic Inc.; or RMI®, Edwards Lifesciences LLC, Irvine, CA, USA) and 17–28 Fr venous cannulae (DLP®, Bio-Medicus, Medtronic Inc.; or RMI®, Edwards Lifesciences LLC).

### ECMO management

Because the cardiac catheterization laboratory is located next to the ED, all candidates for ECMO were relocated to the catheterization laboratory. Here, ECMO cannulation was performed for all patients via the femoral artery and femoral vein using the Seldinger method following an intravenous injection of 50–80 U/kg heparin. Heparin was administered at 300–1400 U/h under the maintenance of activated clotting time (ACT) at 140–180 s from January 2006 to September 2008; from October 2008 onward, nafamostat mesilate (SK Chemicals Life Science Biz., Seoul, Korea Licensed by Torii Pharmaceutical Co. Ltd, Tokyo, Japan) was administered at 0.4–1.5 mg · kg^−1^ · h^−1^ under the maintenance of partial thromboplastin time at 60–80 s [11].

All patients underwent percutaneous coronary intervention (PCI) after ECMO. The ECMO flow was maintained at 3.0–4.0 L/min, while the mean blood pressure was sustained at >60 mmHg. If necessary, norepinephrine or dopamine was also administered. On the day of PCI, clopidogrel and aspirin were administered at doses of 300 mg and 250 mg, respectively; from the second day onward, the doses were 75 mg and 100–200 mg, respectively. Care was taken to maintain the hematocrit level and platelet count at 35% and 80,000/μL, respectively, and transfusion was performed when these values decreased. ECMO was removed when the ejection fraction exceeded 30% at the ECMO flow of 1 L/min on echocardiography. Successful ECMO weaning and survival were defined as cases of survival >24 h and >30 d, respectively, after the removal of ECMO.

### Statistical analysis

Statistical analyses were performed using IBM SPSS Statistics (ver. 21; IBM Co., NY, USA); continuous and categorical variables were analysed by the Mann Whitney *U*-test and the Pearson chi square test or Fisher’s exact test, respectively. The univariate and multivariate stepwise logistic regression analysis model was used to identify independent survival-related factors. Values of p < 0.05 were considered statistically significant. Survival outcomes of the patients who survived CA were analysed using the Kaplan-Meier survival method.

## Results

### Patient profiles

To evaluate the efficacy of ECPR for AMI with refractory cardiac arrest, the patients were devided into 2 groups based on whether survived >30 days after the removal of ECMO or not. Among the baseline clinical characteristics of patients, no statistically significant intergroup differences were observed in gender; past medical history; smoking history; pre-ECMO laboratory findings of creatinine, total bilirubin, creatine kinase-MB, myoglobin, troponin-I, lactate; Sequential Organ Failure Assessment score; and the transfusion volume of cryoprecipitate and platelet concentrates. In contrast, statistically significant intergroup differences were observed in patient age, pre-ECMO laboratory findings on blood urea nitrogen, duration of ECMO, and Simplified Acute Physiology Score II (SAPS II) score. ECMO weaning was possible in 12 (32%) of 34 patients, seven (19%) who survived >30 days and five (14%) who were discharged. Of the seven surviving cases (86%), six were classified as Cerebral Performance Category (CPC) Grade 1 and one patient (14%) as CPC Grade 4 (Table 1).

**Table 1:** **Comparison of baseline clinical characteristics between non-survivors and survivors**

	Non-survivors	Survivors	p
	n = 30	n = 7	
Age, years, mean (SD)	63.2 (10.9)	53.7 (10.2)	0.049
Gender, male	20 (67%)	7 (100%)	0.155*
BMI, mean (SD)	24.2 (2.8)	22.5 (1.3)	0.151
Past medical history			
Hypertension	18 (60%)	2 (29%)	0.212*
Diabetes	19 (63%)	2 (29%)	0.202*
Cerebral vascular accident	3 (10%)	1 (14%)	1.000*
Dyslipidemia	3 (10%)	2 (29%)	0.233*
Previous PCI	1 (3%)	-	1.000*
Smoking	14 (47%)	4 (57%)	0.693*
Pre-ECMO laboratory findings, mean (SD)			
BUN	21.69 (7.47)	14.74 (4.65)	0.019
Cr	1.55 (0.77)	1.16 (0.05)	0.065
TB	0.73 (0.35)	0.81 (0.30)	0.474
CK-MB	80.86 (200.18)	5.31 (4.40)	0.983
Myoglobin	2553.63 (7678.72)	379.12 (657.90)	0.424
Troponin-I	22.02 (54.70)	0.28 (0.30)	0.751
pH	6.95 (0.15)	7.00 (0.15)	0.345
Lactate	10.15 (4.36)	7.73 (2.79)	0.448
BNP	734.67 (1177.84)	17.40 (15.37)	0.008
ECMO duration, h, mean (SD)	94.63 (86.20)	156.71 (58.99)	0.029
SOFA score, mean (SD)	15.10 (1.54)	13.86 (1.07)	0.051
SAPS II score, mean (SD)	92.43 (7.95)	80.14 (6.36)	0.001
Daily blood transfusion during ECMO, units/day, mean (SD)			
Packed red blood cell	6.36 (6.04)	2.26 (3.52)	0.002
Fresh frozen plasma	5.00 (4.96)	1.72 (3.04)	0.006
Cryoprecipitate	1.44 (2.61)	1.43 (2.43)	0.635
Platelet concentrate	2.97 (2.83)	2.99 (2.77)	0.747
Outcomes			
Weaning success	5 (17%)	7 (100%)	
Discharged	-	5 (71%)	
CPC grade			
1	-	6 (86%)	
4	-	1 (14%)	

BMI, body mass index; PCI, percutaneous coronary intervention; VT/VF, ventricular tachycardia/ventricular fibrillation; PEA, pulseless electrical activity; ECMO, extracorporeal membrane oxygenation; BUN, blood urea nitrogen; Cr, creatinine; TB, tuberculosis; CK-MB, creatine kinase-MB; BNP, brain natriuretic peptide; CRRT, continuous renal replacement therapy; SOFA, Sequential Organ Failure Assessment; SAPS II, Simplified Acute Physiology Score II; CPC, Cerebral Performance Category.

*Fisher’s Exact test.

### CPR-related and coronary angiographic characteristics

Fourteen (38%) patients underwent out-of-hospital CPR. There was a statistically significant difference between the two groups in door-to-ECMO time and use of intra-aortic balloon pump (IABP), although no statistically significant intergroup differences were found in the status on arrival, transit time from scene of CA to the hospital for out-of-hospital CA, CPR time, ROSC > 20 min during CPR, door-to-balloon time, post-ECMO return of spontaneous beat (ROSB), and CPR-related complications (Table 2).

**Table 2:** **Comparison of CPR-related characteristics between non-survivors and survivors**

	Non-survivors	Survivors	p
	n = 30	n = 7	
Status on arrival			0.395**
Unstable vital sign	7 (23%)	-	
Cardiogenic shock	11 (37%)	2 (29%)	
VT/Vf	10 (33%)	4 (57%)	
PEA	1 (3%)	1 (14%)	
Asystole	1 (3%)	-	
CPR location			0.080*
Out of hospital	9 (30%)	5 (71%)	
In-ED/Cath lab	21 (70%)	2 (29%)	
Transit time from Victims scene to hospital of OHCA	18.0 ± 17.9	22 ± 18.7	0.643
CPR time, min, mean (SD)	52.23 (37.18)	44.71	0.635
ROSC >20 min during CPR	9(30%)	1(14%)	0.647*
Door to balloon time, min, mean (SD)	107.44 (60.61)	66.14 (21.06)	0.054
Door to ECMO time, min, mean (SD)	94.10 (56.96)	42.86 (14.53)	0.001
ROSB After ECMO	28 (93%)	7 (100%)	1.000*
IABP			0.032**
Pre-/with ECMO	10(33%)	-	
After ECMO removal	-	1(14%)	
CPR-related complications			0.181**
Hemothorax	3 (10%)	-	
Chest wall compartment	4 (13%)	-	
Hypoxic brain damage	9 (30%)	2 (29%)	
Pulmonary haemorrhage	5 (17%)	-	

CPR, cardiopulmonary resuscitation; ED, emergency department; Cath lab, catheter laboratory; ECMO, extracorporeal membrane oxygenation; ROSB, return of spontaneous beat. ROSC time is not excluded.

*Fisher’s Exact test; **Pearson chi-square test.

Among the coronary angiography findings, no statistically significant intergroup differences were seen in the number of lesions, location of infarction-related arteries, or PCI type (Table 3).

**Table 3:** **Comparison of angiographic characteristics between non-survivors and survivors**

	Non-survivors	Survivors	p
	n = 30	n = 7	
Coronary angiographic findings			0.157**
Spasm	3 (10%)	-	
Left main	1 (3%)	2 (29%)	
One vessel	6 (20%)	-	
Two vessels	11 (37%)	3 (43%)	
Three vessels	9 (30%)	2 (29%)	
Infarction-related artery			0.285**
Left main	8 (27%)	4 (57%)	
Left anterior descending	10 (33%)	1 (14%)	
Right coronary artery	12 (40%)	2 (29%)	
Percutaneous coronary intervention			0.302**
Stent	19 (63%)	7 (100%)	
Balloon	5 (17%)	-	
Failed	2 (7%)	-	
No procedure	4 (13%)	-	

Three patients showed spontaneous reperfusion in the coronary artery spam during the no-procedure period, but the procedure could not be pursued in one patient who had received bronchial artery embolization because of a continuing massive pulmonary hemorrhage.

**Pearson chi-square test.

### Complications and cause of death

Of the patients who survived, five (71%) developed pneumonia, three (43%) developed bed sores, and two patients each (29%) developed cannula-related complications and acute renal failure. The major causes of death were myocardial dysfunction (n = 10; 33%) and CPR-related complications such as hypoxic brain damage, chest wall compartment syndrome, hemothorax, and pulmonary hemorrhage (n = 12; 40%). The other causes of death were sepsis in four patients, systemic inflammatory reaction syndrome in two, intracranial hemorrhage in two, and multi-organ failure in one patient.

### Univariate and multivariate analysis of independent factors of survival

The univariate analysis performed to determine the independent patient survival-related factors demonstrated statistical significance for door-to-ECMO time ≤60 min (odds ratio [OR], 19.714; 95% confidence interval [CI], 2.017–192.70; p = 0.010), pre-ECMO BUN (OR, 0.834; 95% CI, 0.701–0.992; p = 0.040), and SAPS II score ≤85 (OR, 19.714; 95% CI, 2.017–192.70; p = 0.010). However, in multivariate analysis, statistical significance was observed only for door-to-ECMO time ≤60 min (OR, 6.0; 95% CI, 1,177–852.025; p = 0.033) (Table 4).

**Table 4:** **Univariate analysis and multiple logistic regression analysis of predictors for survival**

Variables	Univariate analysis			Multivariate analysis		
	OR	95% CI	p	OR	95% CI	p
Door-to-ECMO time ≤60 min	19.714	2.017–192.702	0.010	6.000	1.177–852.025	0.033
Pre-ECMO BUN	0.834	0.701–0.992	0.040	0.756	0.563–1.015	0.063
SAPS II score ≤85	19.714	2.017–192.702	0.010	32.802	0.988–1088.519	0.051

ECMO, extracorporeal membrane oxygenation; BUN, blood urea nitrogen; SAPS II, Simplified Acute Physiology Score II; OR, odds ratio; CI, confidence interval.

**Figure 2:**
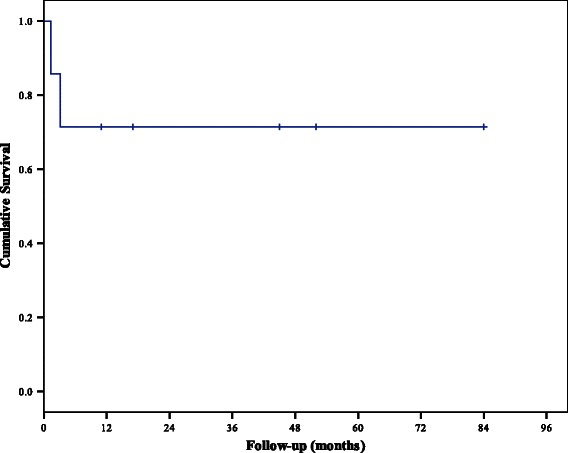
**Kaplan-Meier survival curve.** All five patients considered fit for discharge survived.

Of the seven survival cases, two patients died on the 50^th^ and 94^th^ days, and all five patients who were discharged (CPC Grade 1) survived a median of 45 (range, 11–84) months without the occurrence of serious complications on post-ECMO follow-up (Figure 2).

## Discussion

The survival rates of the patients with out-of-hospital AMI-induced CA who received emergency PCI after a post-CPR ROSC were 38–86% according to inclusion criteria. This is clear evidence of the importance of promptly opening a blocked coronary artery after ROSC in patients with AMI-induced CA [1-3,12]. However, in the hypoxic state caused by the reduced perfusion of other organs during CA, it is difficult to restore the functions of other damaged organs using cardiac reperfusion alone as long as cardiac function is not sufficiently restored. Although this complication may be addressed by the use of large amounts of inotropic agents or an IABP, patients with serious conditions are likely to develop metabolic acidosis due to decreased systemic perfusion, which eventually results in a high mortality rate.

The use of ECMO is highly effective in treating such patients [5,8,13,14]. If ECMO is performed in combination with IABP, IABP-induced pulsatility reduces coronary vascular resistance, thereby increasing coronary blood flow and graft flow, and contributing to the recovery of cardiac function [15,16]. However, in our study, eight patients who received pre-ECMO IABP died, presumably because the lapse of time for IABP insertion and procedure as well as response observation had an adverse effect on the deteriorated systemic perfusion caused by the seriously reduced cardiac function, thus prolonging the state of reduced tissue perfusion and not contributing to the patients’ recovery. In out-of-hospital CA cases induced by AMI, the procedure most helpful for the patients’ recovery appears to be the prompt insertion of ECMO on arrival at the ED to restore tissue perfusion, followed by opening the blocked coronary artery and reducing the amount of inotropic agents administered while ECMO is maintained. Therefore, if a patient with AMI and unstable vital signs or cardiogenic shock arrives at the ED, the prompt use of ECMO should be considered meanwhile the necessary tests, clinical observation, and diagnostic procedures are conducted because of possible CA. This prompt action will avoid time loss during intra-CPR ECMO insertion.

Some studies have found that the shorter the CPR duration during ECPR in CA cases, the higher is the survival rate and the better are the neurologic outcomes [17]. Furthermore, ECPR is more effective than conventional CPR [10], and patients with in-hospital CA have a better chance of survival than patients with out-of-hospital CA [18]. Our study did not find a statistically significant difference in the patients’ CPR time because of the ECMO implantation protocol. However, the door-to-ECMO time was less than 90 min in all survival cases. The survival of AMI-induced CA was influenced by the duration of cardiogenic shock, including CPR time until ECMO implantation from the moment of arrival at the ED, which is the reason why the predictor of survival door-to-ECMO time ≤ 60 min. The patients who developed CA in the ED or catheter laboratory had a relatively high ECMO weaning rate of 32% (7/22); however, their survival rate was as low as 9% (2/22). In the case of five patients who died after ECMO weaning, their cardiac function had been restored. However, the delayed recovery of cardiac function because of pulmonary edema secondary to CPR and cardiogenic shock at the time of admission in combination with concurrent pneumonia (which prolonged the ECMO maintenance time for lung support) ultimately resulted in multiple-organ dysfunction caused by ECMO-related systemic inflammatory response syndrome with increased plasma concentrations of inflammatory cytokines [19]. To reduce the duration of ECMO, which is prolonged by secondary acute lung injury, it is necessary to intensively treat the CPR-induced pulmonary edema or hemorrhage. If pulmonary edema occurs because of insufficient draining of the venous cannula, care should be taken to ensure that it does not develop into pneumonia by reducing the occurrence of left atrial (LA) pressure-induced pulmonary edema parallel to the recovery of left ventricular (LV) function. This can be achieved by LA decompression with LA or LV venting through cannula insertion or septostomy [20-22].

Eight patients in the current study died within 24 h of CPR-induced hemothorax, pulmonary hemorrhage due to bronchial artery rupture, or chest compartment syndrome. Of the CPR-related complications, the conditions of the patients who developed hemothorax were very serious with severe bleeding, so we did not attempt any surgical treatment. However, considering one recent report [23], in which good results were obtained from surgical treatment, it seems recommendable to address hemothorax with a more active surgical approach. Furthermore, preventing transfusion-related acute lung injuries can contribute to enhancing patients’ survival rates, when used during intra-CPR catheterization to avoid blood loss by diminishing cannula site bleeding [11,24].

When considering the use of ECMO in patients with AMI and CA, the reduction the CPR duration, and thus the reduction of the aforementioned CPR-related complications, is important to improve the survival rate. Further, the combination of ECMO, and myocardial reperfusion via PCI can ensure good tissue perfusion at an earlier stage.

The major limitations of this study are that it was retrospective in nature and included a small number of patients, and that a comparative study with AMI patients with cardiac arrest who went through conventional treatment wasn’t done. Therefore, a prospective study that includes a group of patients with AMI and CA is required to investigate a target patient group for the application of ECPR.

## Conclusions

We conclude that ECMO insertion within 60 min of the arrival of patients with AMI and CA at the ED appears to be a good option for maintaining myocardial and systemic perfusion, thereby increasing the survival rate of these patients.
